# Expanded Gene Regulatory Network Reveals Potential Light-Responsive Transcription Factors and Target Genes in *Cordyceps militaris*

**DOI:** 10.3390/ijms251910516

**Published:** 2024-09-29

**Authors:** Paradee Buradam, Roypim Thananusak, Mattheos Koffas, Pramote Chumnanpuen, Wanwipa Vongsangnak

**Affiliations:** 1 Department of Zoology, Faculty of Science, Kasetsart University, Bangkok 10900, Thailand; paradee.bur@ku.th; 2 Kasetsart University International College (KUIC), Kasetsart University, Bangkok 10900, Thailand; 3 Omics Center for Agriculture, Bioresources, Food and Health, Kasetsart University (OmiKU), Bangkok 10900, Thailand; roypim.tha@ku.th; 4 Interdisciplinary Graduate Program in Bioscience, Faculty of Science, Kasetsart University, Bangkok 10900, Thailand; koffam@rpi.edu; 5 Department of Chemical and Biological Engineering, Rensselaer Polytechnic Institute, Troy, NY 12180, USA

**Keywords:** *Cordyceps militaris*, transcriptome analysis, light, transcription factor, light-responsive genes

## Abstract

*Cordyceps militaris*, a fungus widely used in traditional Chinese medicine and pharmacology, is recognized for its abundant bioactive compounds, including cordycepin and carotenoids. The growth, development, and metabolite production in various fungi are influenced by the complex interactions between regulatory cascades and light-signaling pathways. However, the mechanisms of gene regulation in response to light exposure in *C. militaris* remain largely unexplored. This study aimed to identify light-responsive genes and potential transcription factors (TFs) in *C. militaris* through an integrative transcriptome analysis. To achieve this, we reconstructed an expanded gene regulatory network (eGRN) comprising 507 TFs and 8662 regulated genes using both interolog-based and homolog-based methods to build the protein–protein interaction network. *Aspergillus nidulans* and *Neurospora crassa* were chosen as templates due to their relevance as fungal models and the extensive study of their light-responsive mechanisms. By utilizing the eGRN as a framework for comparing transcriptomic responses between light-exposure and dark conditions, we identified five key TFs—homeobox TF (CCM_07504), FlbC (CCM_04849), FlbB (CCM_01128), C6 zinc finger TF (CCM_05172), and mcrA (CCM_06477)—along with ten regulated genes within the light-responsive subnetwork. These TFs and regulated genes are likely crucial for the growth, development, and secondary metabolite production in *C. militaris*. Moreover, molecular docking analysis revealed that two novel TFs, CCM_05727 and CCM_06992, exhibit strong binding affinities and favorable docking scores with the primary light-responsive protein CmWC-1, suggesting their potential roles in light signaling pathways. This information provides an important functional interactive network for future studies on global transcriptional regulation in *C. militaris* and related fungi.

## 1. Introduction

*Cordyceps militaris*, a fungus from the Ascomycetes class, is known for its entomopathogenic nature. This species, notable within the *Cordyceps* genus, typically infects the larvae of lepidopteran insects and produces fruiting bodies on its insect hosts [1]. Traditionally used in Chinese medicine, *C. militaris* is valued for its numerous bioactive compounds, including cordycepin, carotenoids, adenosine, amino acids, ergosterol, polysaccharides, superoxide dismutase (SOD), organic selenium, and a variety of vitamins [2]. These compounds contribute to its significant potential in industrial applications.

*C. militaris* is capable of growing in both solid and liquid media, utilizing a variety of carbon and nitrogen sources [3]. Its growth and biological production are affected by various factors, such as media type, pH, temperature, incubation time, and culture conditions [4,5]. The cultivation process of *C. militaris* includes three main stages: mycelium colonization, primordial initiation, and fruiting body formation. Light becomes essential after the primordial initiation stage because it triggers the formation of the fruiting body [6]. Additionally, light impacts growth and the biosynthesis of secondary metabolites [7].

Recent research has investigated the effects of light on growth and metabolite production in *C. militaris*, which can detect light through photoreceptors. White-collar (WC) proteins and phytochromes are two families of proteins that sense short-wavelength and long-wavelength light, respectively. These photoreceptors regulate the expression of genes involved in development, stress response, secondary metabolite biosynthesis, and the circadian clock [8]. For example, white collar-1 (WC-1) contains zinc finger domains that activate Zn(II)2Cys6-type transcription factors (TFs), essential for cellular metabolism, sexual and asexual development [9], and the biosynthesis of secondary metabolites like cordycepin and carotenoids [10].

*C. militaris* has been extensively studied using omics technologies, including genomics [11] and transcriptomics [12]. High-throughput technologies have been employed to explore gene regulation, biosynthesis, and metabolic pathways. To understand these processes at a systems level, integrating biological knowledge with large-scale data is crucial. Recently, the metabolic responses of carotenoid and cordycepin biosynthetic pathways in *C. militaris* under light exposure were elucidated. Genes associated with cordycepin biosynthesis were upregulated under light conditions compared to dark conditions [13]. Additionally, some transcription factors have been found to co-regulate a large group of growth- and developmental-involved genes [14]. However, the mechanisms of gene regulation under light exposure remain largely unexplored.

Thus, this study aimed to identify light-responsive genes and potential transcription factors of *C. militaris* using integrative transcriptome analysis. To obtain informative data, we reconstructed the expanding gene regulatory network (eGRN) of *C. militaris* by using both interolog-based and homolog-based methods to build the protein–protein interaction network. *A. nidulans* and *N. crassa* were chosen as templates due to their relevance as fungal models and their close relationship to *C. militaris*. Then, transcriptome analysis was conducted through *C. militaris* cultivation towards differentially expressed genes (DEGs), comparing light-exposure and dark conditions. Taken together, the eGRN was used as a scaffold for integrative transcriptome analysis to identify light-responsive genes and the potential transcription factors of *C. militaris*. The useful information may be helpful for further global transcriptional regulation studies in *C. militaris* and related fungi at the systematic level.

## 2. Results and Discussion

### 2.1. The Reconstructed eGRN of C. militaris

**Figure 1:**
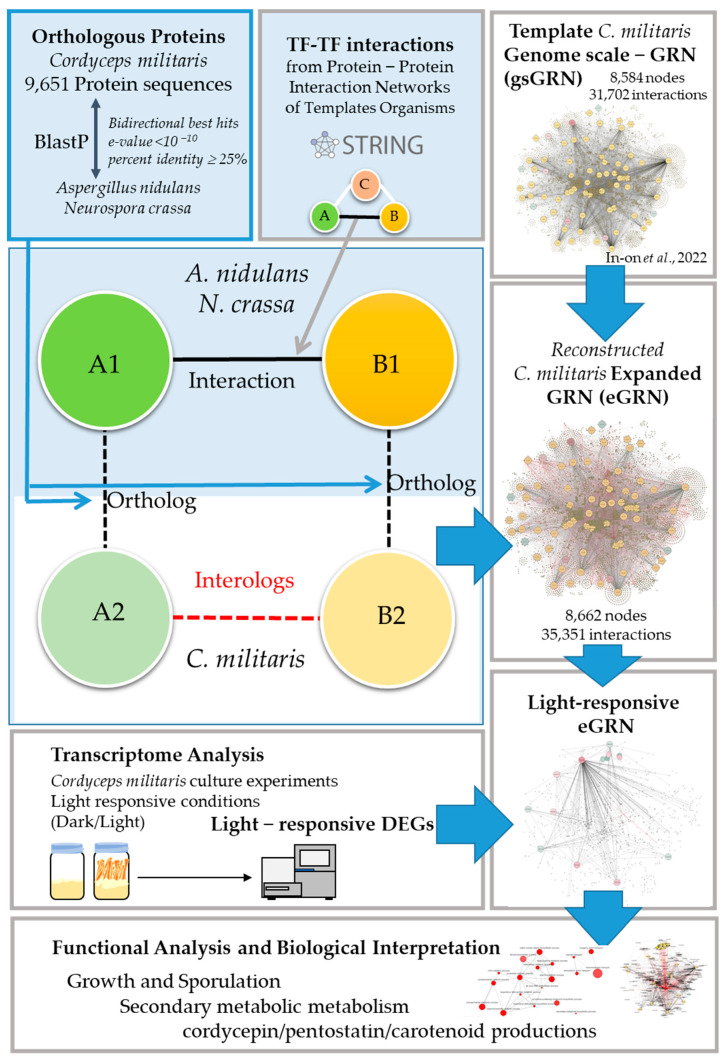
The overall workflow of eGRN reconstruction of *C. militaris* and functional transcriptomic analysis to reveal the light-responsive transcription factors and target genes. Nodes A, B, and C serve as examples, representing proteins and genes involved in interactions.

The pipeline for the reconstruction of the eGRN of *C. militaris* is illustrated in Figure 1.

**Figure 2:**
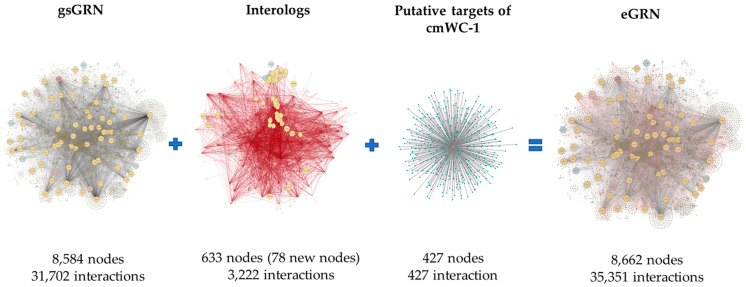
The reconstructed eGRN integratively merged from genome-scale GRN (gsGRN), interologs, and putative targets of cmWC-1. The red lines indicate interologous interaction edges from orthologous pairs adopted from the *A. nidulans* and *N. crassa* PPI networks.

**Figure 3:**
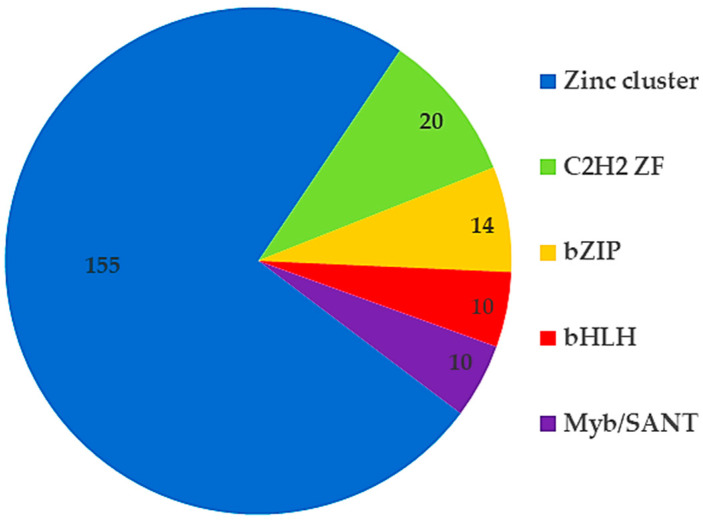
The pie chart represents the top five transcription factors families in eGRN of *C. militaris*. The numbers in each section of the pie chart indicate the number of transcription factors belonging to specific clusters.

Initially, the interolog-based method was used to construct the protein–protein interaction network of *C. militaris*. Based on the homology-based principle of the method, *A. nidulans* and *N. crassa* were selected as templates, based on the model of fungi and closely related to *C. militaris*. According to protein–protein interaction data, *A. nidulans* has 35,724 interactions of 2639 proteins and *N. crassa* has 38,352 interactions of 2644 proteins. These data were employed to predict the protein–protein interaction network of *C. militaris* based on the functional conservation assumption of the orthologous protein. The *C. militaris* orthologous protein of each known protein–protein interaction was searched by BLASTp based on the following criteria: sequence identity ≥ 25%, E-value < 10^−10^ and bidirectional best hits (BBHs). The interologous TFs-TFs network consisted of 3222 interactions of 633 nodes (including 78 new nodes). This interologous TFs-TFs network, together with the additional 427 putative TFs-regulated genes of CmWC-1 (CCM_01180) from the literature [15], was integrated into the previous genome-scale GRN (gsGRN) [14]. Accordingly, the reconstructed eGRN contained 35,351 putative interactions, distributed into 507 TFs and 8662 regulated genes (Figure 2). Among these TFs, the top five TFs families were identified—for example, zinc cluster, C2H2 Zinc finger, bZIP, bHLH, and Myb/SANT (Figure 3). The TFs family with the highest number of TFs (155 TFs) was the Zn cluster. The Zn cluster family has many related functions, such as sugar and amino acid metabolism, gluconeogenesis, respiration, vitamin synthesis, cell cycle, chromatin remodeling, nitrogen utilization, peroxisome proliferation, drug resistance, and stress response [16]. Furthermore, this family of TFs is important for fruiting body development [11] and secondary metabolites in *C. militaris* [10].

### 2.2. Growth Characteristics and Production of Cordycepin and Carotenoids

The growth characteristics of the *C. militaris* strain TBRC6039 were investigated in a defined medium under different light conditions. When cultivated in the dark for 14 days, the fungus produced mycelia. However, upon exposure to light for an additional 28 days, fruiting body formation was observed, accompanied by a change in color from white to yellow-orange. The biomass of *C. militaris* increased over time, with the highest total biomass content recorded under light conditions. These results are consistent with previous studies that have also reported the light-induced nature of fruiting body development and carotenoid production in *C. militaris* [17].

Interestingly, the contents of cordycepin and carotenoids also increased concomitantly with the biomass, reaching their maximum levels under light exposure. The cordycepin content was 23.294 ± 0.80 mg/g cell mass under light conditions, which was 1.8 times higher than that observed in the dark. Moreover, the carotenoid content under light conditions (1.916 ± 0.09 mg/g cell mass) was significantly higher compared to dark conditions (Table 1). These findings are consistent with a study that investigated the inductive effect of light on cordycepin and adenosine productions [6,13]. The overproduction of cordycepin and carotenoids appears to be closely linked to the light-induced fruiting body formation in *C. militaris*. This relationship has been observed in other studies, where light was found to be an essential environmental factor for *C. militaris* primordial initiation and fruiting body growth [18]. Although carotenoids may not be directly involved in fruiting body formation, they could act as free radical scavengers, helping protect the fungus from oxidative stress during this crucial developmental stage [19].

*C. militaris* may use a light-induced stress response mechanism during fruiting body formation to optimize the production of valuable secondary metabolites, such as cordycepin. This hypothesis is supported by our observation that the highest total biomass and levels of cordycepin and carotenoids were achieved under the light condition. This suggests that adjusting cultivation conditions to include light could enhance the production of these valuable metabolites.

### 2.3. Comparative Transcriptome Analysis of C. militaris under Dark and Light Conditions

To investigate the total expressed genes, in this study, *C. militaris* samples in two biological replicates in the dark condition (D) and light condition (L) were sequenced using an Illumina NovaSeq 6000 system. As shown in Table 2, total clean reads were retrieved with an average sequencing depth of 22.89 ± 1.76 million paired-end reads, with a %GC average of 56.25 ± 0.39. The percentage of uniquely mapped reads of 95.76 ± 0.19 was obtained based on the *C. militaris* CM01 genome [11].

**Figure 4:**
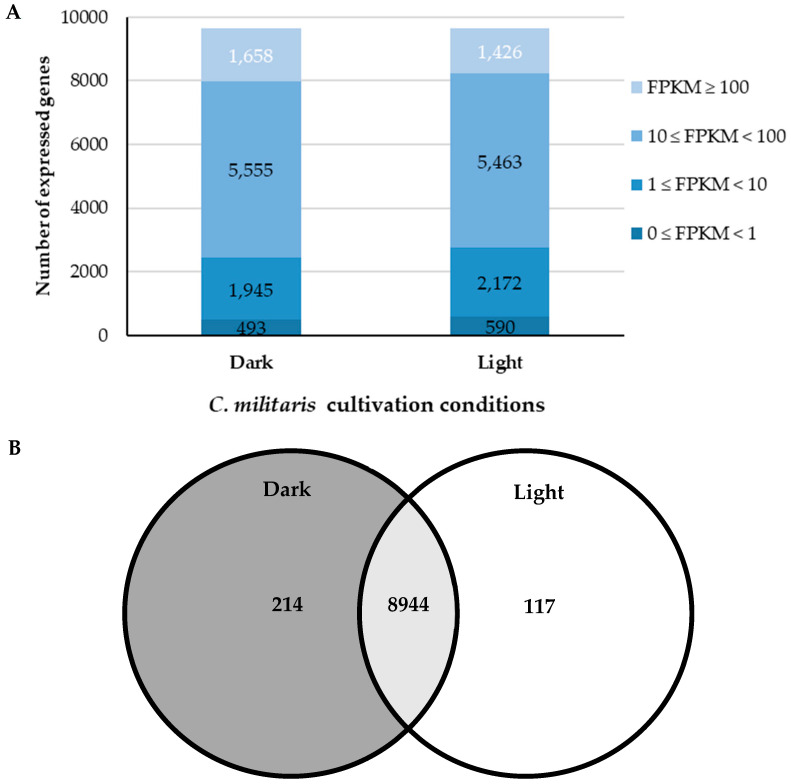
Gene expression levels from comparative transcriptome of *C. militaris*. (**A**) Distribution diagram shows genes with different FPKM values across the two conditions, (**B**) Venn diagram shows expressed genes of each condition. The number of genes in the overlapping area represents the genes expressed in both conditions.

The analysis of transcriptome data showed the distribution of FPKM values across all genes (Figure 4A). The mean FPKM of the two biological replicates was calculated for each condition using the geometric normalization method. All genes were classified into four categories according to their FPKM values. Mostly, the genes with moderate expression levels (10 ≤ FPKM < 100) were found in both D and L conditions. Notably, the D condition had a greater number of highly expressed genes (FPKM ≥ 100), which could be simply explained by the fact that the D condition was a more active growth stage, while the L condition had a greater number of less expressed genes (1 ≤ FPKM < 10).

Genes with a mean of FPKM ≥ 1 in at least one stage were considered to be expressed, resulting in 9275 expressed genes used for subsequent analysis. In total, 9158 and 9061 genes were expressed in the D and L conditions, respectively. As shown in Figure 4B, a total of 8944 genes was expressed across the two conditions.

### 2.4. Identified Differentially Expressed Genes (DEGs)

**Figure 5:**
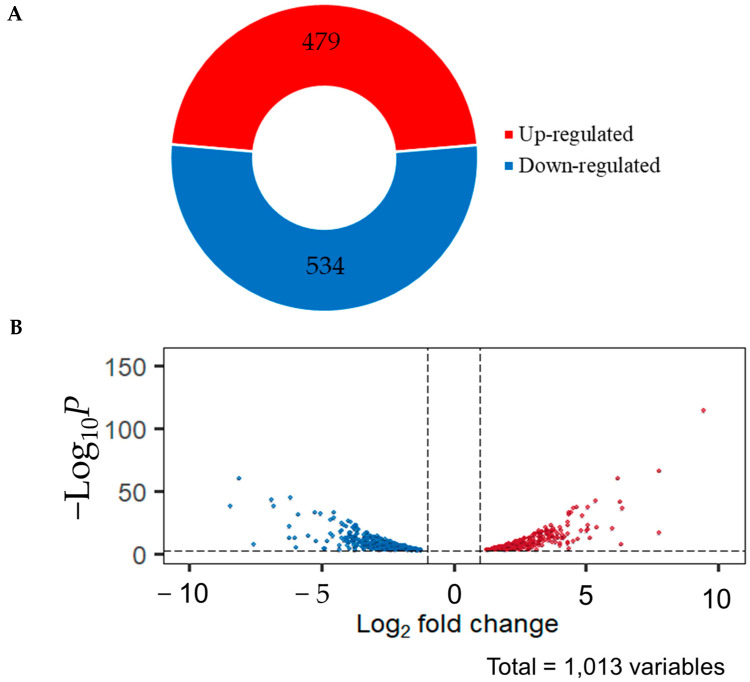
Differentially expressed genes (DEGs) of *C. militaris*. (**A**) The pie chart represents the number of DEGs between the dark and light conditions. (**B**) Volcano plot of differentially expressed genes was identified between the light condition and the dark condition. The blue dots represent downregulated genes, and the red dots represent upregulated genes. The black dash lines indicate the significance cut off zones.

To identify DEGs, all expressed genes were compared based on the DEGs analysis by DESeq2 using |log2fold change| ≥ 1 and FDR < 0.001 as the threshold. The DEGs analyzed data showed that there are 1013 DEGs, of which 479 genes were upregulated in light conditions and 534 were downregulated genes (Figure 5). All DEGs were used for mapping to the eGRN to extract the condition-specific gene regulatory network.

**Figure 6:**
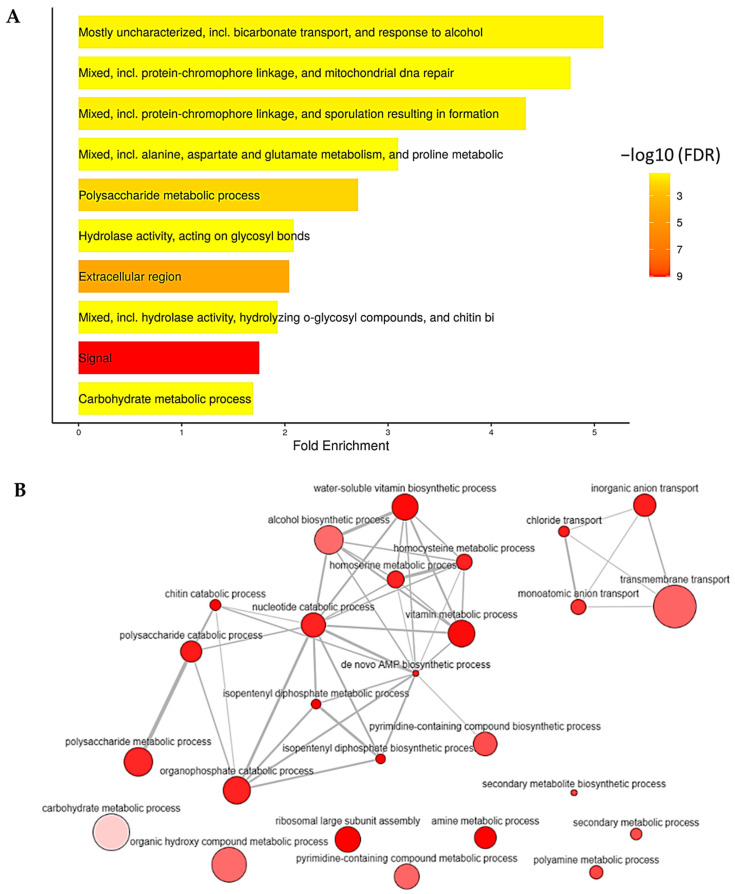
Gene Ontology (GO) enrichment analysis based on all light-responsive differentially expressed genes (DEGs) of C. militaris. (**A**) The bar plot represents the enrichment false discovery rate (FDR) and fold change of DEGs between dark and light conditions. Yellow and red shading indicate the FDR values. (**B**) The GO enrichment network shows the relationships between enriched pathways. Node size represents the number of proteins associated with each GO term, and a higher intensity of the red color indicates greater significance (based on *p*-value).

All significant genes were annotated based on the KEGG database and classified into 10-top function categories, as illustrated in Figure 6A. In the metabolism category, the most genes are involved in amino acid metabolism, carbohydrate metabolism, secoundary metabolic process and lipid metabolism, respectively (Figure 6B), in light-exposure conditions.

### 2.5. Identified Light-Responsive Genes and Potential TFs Using the Reconstructed eGRN

**Figure 7:**
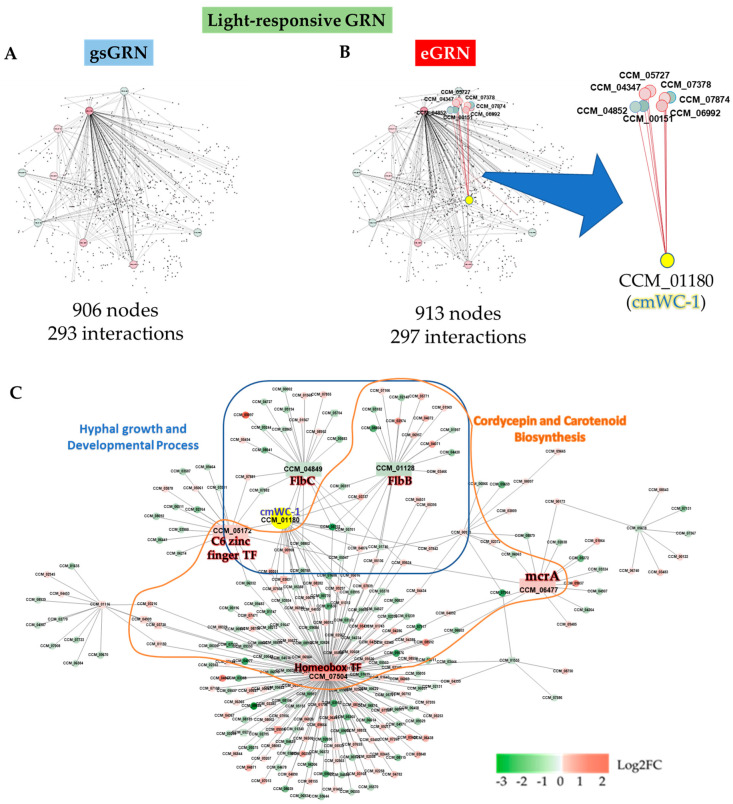
Light-responsive GRN; (**A**) genome-scale GRN and (**B**) expanded GRN after mapping with DEGs (the red and green node colors indicate the gene expression level). (**C**) A light-responsive subnetwork of TFs-regulated genes of *C. militaris* (Supplementary Table S1). The rectangle nodes represent five key light-responsive transcription factors, and the circle nodes shows the regulated genes (the red and green node colors indicate the gene expression level). The blue line indicates the genes involved in growth (hyphal growth and development processes), while the orange line indicates those genes involved in the biosynthesis of secondary metabolites (cordycepin and carotenoid biosynthesis).

**Figure 8:**
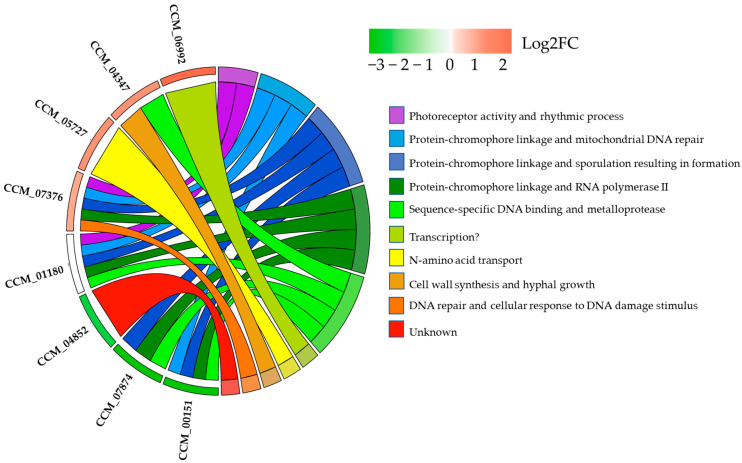
GO chord plot. Chord plot showing significantly enriched biological process GO terms of seven target genes of CMM_01180 (CmWC-1). The left of the plot shows the genes contributing to the enrichment, arranged in order of their log2FC, which is displayed in descending intensity of red squares for the upregulated genes and green squares for the downregulated ones. The genes are linked to their assigned terms via colored ribbons.

To identify light-responsive genes and potential TFs, we firstly used the eGRN as a scaffold, before integrating gene-level statistics (e.g., *p*-values) from a set of DEGs in response to light. As a result, a light-responsive subnetwork of TFs-regulated genes containing 273 nodes and 295 interactions was illustrated using cytoscape, as shown in Figure 7C. Compared to the DEG mapping results on the genome-scale GRN (Figure 7A), our newly reconstructed eGRN successfully revealed seven target genes of CCM_01180 (CmWC-1) (Figure 7B). Interestingly, all GO terms from the GO enrichment analysis of these target genes (Figure 8) were primarily associated with photoreception, circadian rhythm, sporulation, hyphal growth, and DNA repair. These biological process clusters showed significant linkage to light-response mechanisms related to developmental growth and UV-stress response.

The functions of the CCM_04852 and CCM_06992 gene products are currently unannotated with no specific GO terms available, leaving them classified as hypothetical proteins. To bridge this gap, in silico analysis using HDOCK identified the FHA domain of the transcription factor ArnA from *Sulfolobus acidocaldarius* (PDB ID: 5A8I) as the closest similarity template. Given its role in DNA binding and gene regulation, CCM_04852 is likely involved in transcriptional regulation. Therefore, we propose “transcription” as a potential GO term for this particular gene. However, experimental validation is required to confirm this function and assign the appropriate GO annotations.

As seen in Table 3, the top five TFs with the highest number of regulated genes and highest normalized betweenness scores are CCM_07504, CCM_04849, CCM_01128, CCM_05172, and CCM_06477. The normalized betweenness scores further imply that the homeobox TF is central to the regulatory network, potentially serving as a key hub that integrates various signaling pathways to orchestrate the organism’s adaptive responses to environmental changes. As such, the homeobox transcription factor encoded by gene ID CCM_07504 showed the highest number of regulated genes. The homeobox transcription factor is related to the regulation of sporulation (conidiogenesis) and fruiting body formation, and it may be important for the cordycepin and carotenoid production of *C. militaris* [20,21]. In *A. nidulans*, homeobox transcription factors, particularly HbxA, are essential in regulating gene expression and developmental processes. HbxA controls the expression of over a thousand genes, including those related to development and secondary metabolism, influencing the production of key metabolites like nidulanins and sterigmatocystin. It also plays a critical role in stress response and β-glucan biogenesis. Disrupting HbxA results in major changes in these processes, highlighting its significance in both development and fungal natural product biosynthesis, with potential agricultural and medical implications [21,22,23]. Similarly, in *Neurospora crassa*, homeobox transcription factors are implicated in regulating developmental processes, including the differentiation of reproductive structures and the response to environmental cues, thus demonstrating a conserved function across different fungal species in orchestrating complex life cycles and metabolic pathways [21,24].

This was followed by C2H2 ZF transcription factor encoding by CCM_04849, which showed a high number of regulated genes (21 genes). As previously reported, CCM_04849 is an FlbC ortholog in *A. nidulans* that regulates cell growth and development [25]. FlbC and FlbB are known to regulate conidiation and development in other fungi [26], indicating that light represses these TFs to inhibit asexual reproduction and promote other developmental pathways in *C. militaris*. Considering the others, CCM_01128 is observably a bZIP-type transcription factor (FlbB ortholog) that controls developmental transitions by triggering the production of asexual multicellular structures [27]. FlbB has been related to secondary metabolite synthesis; therefore, CCM_01128 might be important to cordycepin and carotenoid biosynthesis. CCM_05172 is a C6 zinc finger domain-containing protein with regulatory functions in both primary and secondary metabolisms [28]. Moreover, the ortholog of CCM_05172 is involved in the circadian clock in *N. crassa* [29]. Considering CCM_06477, it is an mcrA ortholog. These C6 zinc finger TFs, including mcrA (CCM_06477), are known to regulate secondary metabolism and stress responses in *Aspergillus* and *Penicillium* [30].

As described in the top five TFs, altogether, 240 genes were noticeably regulated, which accounted for 88% in the light-responsive network. These were targets of interest in further investigations in relation to cell growth and development, as well as the biosynthesis of cordycepin and carotenoids in *C. militaris.* In this study, we adopted experimentally validated interactions from the previously published work regarding the transcription profiling and direct target genes of CmWC-1 in *C. militaris* [15] to reconstruct our expanded gene regulatory network (GRN). These interactions provide a solid foundation for identifying key transcription factors (TFs) involved in light-responsive pathways. However, to further validate and refine the proposed network, future experimental methods, such as electrophoretic mobility shift assays (EMSAs), yeast one-hybrid assays (Y1H), and yeast two-hybrid (Y2H) assays, should be instrumental. These alternative approaches would help to verify the interaction relationships within the GRN and deepen our understanding of the regulatory roles these TFs play. This work demonstrates the utility of the eGRN approach in predicting novel TFs in light-responsive networks, offering a strong basis for future experimental validation.

### 2.6. Light-Responsive Genes and Potential TFs Involving Hyphal Growth, Sporulation, Biosynthesis of Cordycepin and Carotenoids in C. militaris

Light plays a crucial role in growth, development, and secondary metabolite production in fungi. As illustrated in Figure 7C, the potential five TFs and target of regulated genes in relation to the growth, development and biosynthesis of cordycepin and carotenoid in *C. militaris* are shown. As described in the following, the homeobox transcription factor directly regulated *veA* (CCM_04531) and *lae* (CCM_05395), which were associated with sexual reproduction. Both genes were significantly upregulated compared to the dark condition. In addition, *fadA* (CCM_07236) and *mat1* (CCM_06523) were directly regulated by the FlbC transcription factor. *fadA* was upregulated, while *mat1* was downregulated. It is shown that light may regulate sexual reproduction, growth, and development in *C. militaris.*

For the biosynthesis of cordycepin and carotenoid, a previous study reported the gene-encoding enzymes associated with cordycepin, i.e., *Cns1*–*Cns4* [31]. They found that *Cns1* and *Cns2* are responsible for cordycepin biosynthesis. *Cns3* and *Cns4* are responsible for pentostatin (PTN) production and exportation, respectively. PTN is an adenosine deaminase inhibitor, which helps to protect cordycepin from deamination. In the current study, even though *Cns1* (CCM_04436), *Cns2* (CCM_04437), *Cns3* (CCM_04438), and *Cns4* (CCM_04439) were involved in cordycepin biosynthesis, there were no significant differences. However, *Cns1*, *Cns2*, and *Cns3* were found to be downregulated, while *Cns4* was upregulated under light exposure compared to dark conditions. Thus, it is predicted that light may induce the production and exportation of PTN. Likewise, *CAO-2* (CCM_06728) and *YLO-1* (CCM_09115) genes were found to be involved in the biosynthesis of carotenoids [32], which were upregulated when comparing light-exposure and dark conditions.

Taken together, the homeobox TF might indirectly regulate *Cns1*–*Cns3*, while *Cns4* directly regulated *Cns4*. FlbB might indirectly regulate *Cns1*, *Cns2,* and *Cns4* but directly regulate *Cns3*. *CAO-2* and *YLO-1* might be regulated by the homeobox TF. Thus, we propose that cordycepin biosynthesis may be inversely coupled with transcription factor functional modules.

According to the mapped TF–gene interactive network, the five key TFs found to regulate the ten genes included *CCM_04531*, *CCM_05395*, *CCM_07236*, *CCM_06523*, *CCM_04436*, *CCM_04437*, *CCM_04438*, *CCM_04439*, *CCM_06728*, and *CCM_09115*. Both the TFs and their target genes are posited to play a pivotal role in the growth, development, and production of secondary metabolites in *C. militaris*.

### 2.7. The Potential Physical Interaction of the CmWC-1 and Its Candidate Protein Targets

Previously, the identification of target genes regulated by CmWC-1, the primary photoreceptor in *C. militaris*, was achieved through ChIP-seq analysis, revealing a complex network of light-responsive gene regulation. A total of 270 significant peaks corresponding to 427 genes were identified, with 143 of these genes specifically responding to light stimuli. The binding site for CmWC-1 was determined to be AAATCAGACCAC/GTGGTCTGATTT, highlighting a specific DNA motif crucial for its regulatory function [15]. Theses 427 target genes were added into our eGRN to connect the regulatory TFs to all possible hierarchical light signal transductions in *C. militaris*. The regulation of gene expression by CmWC-1 occurs primarily through transcriptional mechanisms, where the protein interacts with specific DNA sequences to activate or repress target genes. However, it is essential to recognize that transcriptional regulation is not solely dependent on protein–DNA interactions. Physical protein–protein interactions can also play a significant role in activating regulatory pathways [17,33]. This dual mechanism allows for a more nuanced response to environmental signals, such as light [15].

**Figure 9:**
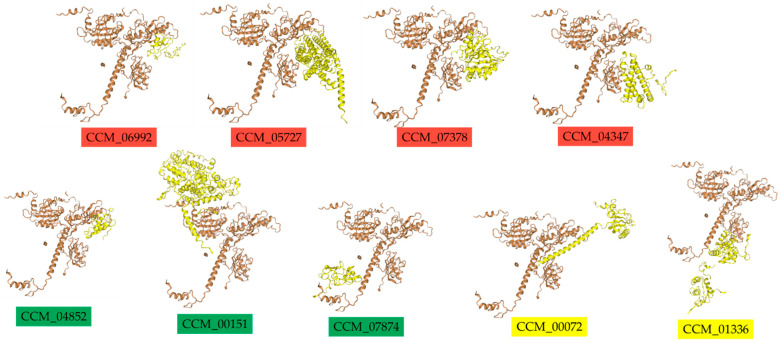
Molecular docking simulations of seven target genes of CMM_01180 (cmWC-1). The docking affinities were compared with two known target proteins, CCM_00072 (cmWC-2) and CCM_01336 (SKN7-like protein), which have been experimentally validated as having the highest and lowest binding scores, respectively. The brown protein structure represents cmWC-1, while the yellow protein structure represents the ligand candidates. The CmWC-1 target gene names are highlighted in red for those upregulated in response to light conditions, green for those downregulated, and yellow for known physical interaction targets.

To further validate the potential physical interactions between CmWC-1 and its target proteins, template-based protein–protein docking simulations using the HDOCK server [34] were performed. These simulations aimed to assess the binding affinities and potential interactions of CmWC-1 with the seven proteins of the identified DEGs. The results from these simulations (Figure 9) can provide insights into how CmWC-1 (CCM_01180) may physically associate with these proteins, potentially influencing their activity and the overall regulatory network in response to light in *C. militaris*. These results were compared with two known target proteins, CCM_00072 (cmWC-2) and CCM_01336 (SKN7-like protein), which have been experimentally validated as having the highest and lowest binding scores, respectively (Figure 9).

Among the seven novel candidates, CCM_05727 (amino acid transporter) exhibits the lowest docking score (−330.6), suggesting the strongest potential binding affinity to cmWC1. This score is even lower than that of CCM_00072 or cmWC-2 (−301.36), which have the highest experimentally determined binding affinity (Table 4). The novel candidates like CCM_06992 also show strong binding potential, with a docking score of −289.5, followed by CPD Photolyase or CCM_00151 (−273.95). The rest of the novel candidates have docking scores ranging from -222.17 (CCM_07874) to −254.37 (CCM_04347), all of which indicate moderate to strong binding potential compared to CCM_01336 (−262.15), which has the lowest experimentally determined score.

According to the docking results in Table 4, the confidence score reflects the reliability of the predicted interactions. CCM_05727 (amino acid transporter) again stands out with the highest confidence score (0.9737) among the novel candidates, indicating a highly reliable prediction. This is followed by CCM_06992 (0.9421) and CCM_00151 (0.9227), which also exhibit strong reliability. These scores are comparable to those of the known targets CCM_00072 (0.9538) and CCM_01336 (0.904). The remaining novel TFs have confidence scores ranging from 0.809 (CCM_07874 or cyanovirin-N) to 0.8897 (CCM_04347 or vegetative cell wall protein gp1), indicating varying degrees of prediction reliability.

The ligand RMSD values provide insights into the conformational stability of the docked complexes. Notably, the unknown protein CCM_06992 demonstrated the lowest RMSD value (38.27 Å), suggesting a more stable interaction with cmWC-1, which could imply a more favorable binding conformation. This is significantly more stable than both CCM_00072 (79.8 Å) and CCM_01336 (79.58 Å). Other novel TFs, such as CCM_04852 (45.19 Å) and CCM_07378 (44.5 Å), also show moderate stability. However, CCM_00151 (65.63 Å) and CCM_05727 (59.91 Å) have relatively higher RMSD values, which might suggest some conformational flexibility, potentially affecting their functional binding.

All TFs were docked using the 4F3L receptor, corresponding to the heterodimeric CLOCK transcriptional activator complex. This receptor is known for its role in circadian rhythm regulation, suggesting that these TFs may play a similar role in light-responsive or circadian-like mechanisms in *C. militaris*.

In conclusion, the docking results suggest that among the novel TFs, CCM_05727 and CCM_06992 are the most promising candidates for interacting with CmWC-1, with strong docking and confidence scores, along with reasonable RMSD values. These TFs could be comparable or even superior to the known target CmWC-2 (CCM_00072). Experimental validation is necessary to confirm these interactions and to further explore their potential roles in light-responsive gene regulation in *C. militaris*.

## 3. Materials and Methods

### 3.1. Expanding Gene Regulatory Network (eGRN) of C. militaris

The earlier genome-scale gene regulatory network (GRN) published by [14], containing 71 TFs and 31,703 interactions, was used as a template for a further expanded GRN (eGRN) throughout this study. To establish an eGRN, we initially constructed TF interaction subnetworks of *C. militaris* using protein orthologs and interolog approaches against *A. nidulans* FGSC A4 proteins using the NCBI database (www.ncbi.nlm.nih.gov/) (accessed on 10 August 2021) and protein–protein networks of *N. crassa* OR74A, according to Search Tool for the Retrieval of Interacting Genes/Proteins (STRING) (string-db.org) as illustrated in Figure 1. Notably, the STRING database imported experimentally derived protein–protein interaction data through literature curation. It is noted that bidirectional best hits (BBHs) analysis using BLASTp was performed under % identity ≥ 25 and E-value ≤ 10^−10^ for protein orthologs. Next, the Fungal Transcription Factor Database (FTFD) [35] and Gene Ontology (GO) databases were used to predict the TFs of *C. militaris*. Moreover, the TF interaction subnetwork of cmWC-1 (CCM_01180) with 427 target regulated genes (containing the binding sites for CmWC-1 in their promoter) was lastly reconstructed to gain the eGRN by literature curation [15].

### 3.2. Transcriptome Analysis of C. militaris

The *C. militaris* strain TBRC6039 was utilized in this study. For the light condition, the strain was cultured in a rice medium composed of an equal ratio of rice grains and defined glucose medium (1:1, *w*/*v*) under static conditions at 22 ± 2 °C. The fungal cultivation was initially conducted in the dark for 14 days, followed by switching light (~1000 lux) and dark conditions every 12 h for a total of 56 days. All cultivation experiments were carried out in three biological replicates. Samples were harvested at various time points to assess growth and measure metabolites. For subsequent transcriptome analysis, samples from the 28-day culture were immediately frozen in liquid nitrogen and stored at −80 °C until RNA extraction. For the dark condition, a previous study reported by [36] was used as a reference.

The fungal samples from the 28-day culture were freeze-dried overnight at −90 °C. The dried samples were then weighed to determine their dry cell weight (DCW) and ground into a fine powder for growth assessment and targeted metabolite analysis, including glucosamine, cordycepin, and carotenoids. Glucosamine content was used as an indirect measure to estimate biomass. The powdered samples were extracted, and glucosamine levels were quantified using a modified method based on a previous study [36]. The glucosamine content was calculated using the following equation:

$$Glucosamine\;content\left(mg\;{gDW}^{-1}\right)=\frac{C\;\times \;V_{f}\;\times \;V_{e}}{W_{e}\;\times \;V_{a}\;\times \;1000}$$

where *C* (µg/mL) is the glucosamine content derived from standard curve, *V_f_* (mL) is the final volume of the extraction solution, *V_e_* (mL) is the the volume of the extraction reagent, *V_a_* (mL) is the volume of the analysis solution and *W_e_* (gDW) is the weight of the dried sample used for extraction.

The fungal biomass was then estimated using the following formula:

$$Biomass\left(g\;{kg}^{-1}\right)=\frac{G\;\times \;W_{f}\;}{W_{s}}$$

where *G* (mg/gDW) is the glucosamine content of the analysis solution, *W_f_* (gDW) is the dry weight of the fermented sample, and *W_s_* (g) is the dry weight of the fermented substrate.

To measure cordycepin content, dried *C. militaris* powder was mixed with water, sonicated for 30 min, and analyzed by high-performance liquid chromatography (HPLC) using a HiQSil C18HS column (300 mm × 4.6 mm, 5 µm) at 40 °C with UV detection at 260 nm. The mobile phase was 15% methanol, with a flow rate of 0.6 mL/min for 25 min. For carotenoid content, they were extracted from the dried powder using an acid-heating method, and quantified colorimetrically by measuring absorbance at 445 nm [37]. Data are presented as mean ± standard deviation (*n* = 3), with statistical significance determined using a Student’s *t*-test (*p* < 0.05).

The harvested cells were immediately immersed in liquid nitrogen for total RNA extraction. The sample (~500 mg) was ground using a cold mortar and pestle and subjected to total RNA extraction and purification using a RNeasy mini kit (Qiagen, Hilden, Germany) according to the instruction manual. The total RNA amount was assessed by measuring absorbance at 260 and 280 nm. The quality of total RNA was determined by gel electrophoresis (Bio-Rad, Hercules, CA, USA), a Nanodrops Spectrophotometer (Thermo Fisher Scientific, Waltham, MA, USA), and an Agilent 2100 bioanalyzer (Agilent, Santa Clara, CA, USA). Subsequently, the qualified total RNA was treated with DNase I to remove genomic DNA, and then magnetic beads coated with oligo-deoxythymidylate (oligo(dT)) primers were used for mRNA isolation. For the construction of the sequencing library, mRNA was mixed with the buffer, and then the fragmented mRNAs were used as templates for cDNA library construction. The library was subsequently connected with paired-end adaptors and sequenced using the Illumina NovaSeq 6000 system (Illumina, San Diego, CA, USA).

Raw RNA-Seq data were kept in FASTQ format, and the FASTQ files, including detailed read sequences, were deposited in the NCBI Sequence Read Archive under the BioProject accession number PRJNA1146582 (BioSample: SAMN43108627 and SAMN43108692) and checked by FastQC [38]. The paired-end reads containing poly-N were filtered, and the adapter sequences were trimmed using the cutadapt program [39] to obtain clean RNA-Seq data. For RNA-Seq mapping, the clean reads were mapped to the *C. militaris* CM01 genome [17] using the STAR two-pass-mode version 2.7.8a [40] for estimating mapped reads to all reference genes. Unique mapped reads with ≥5 mapped reads were counted by STAR using quantMode GeneCounts.

The genes that had mapped reads were subjected to the calculation of FPKM (fragment per kilobase of transcript per million mapped reads), which is a normalized estimation value of gene expression based on RNA-Seq data [41] in which the expressed genes are indicated by an FPKM value ≥ 1. For functional assignment, the Joint Genome Institute via the MycoCosm portal, EuKaryotic Orthologous Groups, the Kyoto Encyclopedia of Genes and Genomes (KEGG), Gene Ontology (GO) databases, and the EggNogs database [42] were used for assigning the functional expressed genes. RNA sequencing reads of *C. militaris* strain TBRC6039 in the dark condition (BioProject: PRJNA832515, BioSample SAMN27864466 and SAMN27864467) was retrieved from a previous study [32]. The DEGs analysis between the light-exposure and dark conditions of *C. militaris* was performed using the DESeq2 R package with a Q-value < 0.05 [43]. Genes with a value of |log_2_fold change| ≥ 1 and a false discovery rate (FDR) < 0.001 were identified as significant light-responsive genes.

The GO enrichment analysis was also performed based on hypergeometric test with a threshold *p*-value of < 0.05. The GO enrichment analysis of the target genes was performed using ShinyGO 0.80 (http://bioinformatics.sdstate.edu/go/) (accessed on 6 August 2022), and *Cordyceps militaris* CM01 was selected as a reference species. Based on the classified local network cluster (STRING) from shinyGO, the GO chord plots were generated to show the relationship between the GO term and the CmWC-1 target genes using the online tool from SRplot webserver (http://www.bioinformatics.com.cn/plot_basic_GOplot_chord_plot_085_en) (accessed on 6 August 2022).

### 3.3. Identification of Light-Responsive Genes and Potential TFs Using the eGRN of C. militaris

To identify light-responsive genes and potential TFs, we firstly used the eGRN as a scaffold; then, gene-level statistics (e.g., *p*-values) from a set of DEGs in response to light were integrated. Moreover, the normalized betweenness centrality score was used to measure the centrality in the network based on the shortest paths, according to Equations (1) and (2).

The betweenness centrality score of node *n*

(1) $$B_{\left(n\right)}=\sum_{i\neq j\neq n\in C}\frac{\sigma_{ij}\;(n)}{\sigma_{ij}}\;,$$

where *σ*_*ij*_ is the total number of shortest paths between node *i* and *j* and *σ*_*ij*_ (*n*) is the number of shortest paths between node *i* and *j* that walk through node *n*. The betweenness score of each node can be normalized as follows.

(2) $$Normalized\;betweenness\;score\;of\;node\;n=\frac{B_{\left(n\right)}-min\;(B)}{max(B)-min(B)},\;$$

where *B*_(*n*)_ is the betweenness centrality score of node *n*, and *max(B)* and *min(B)* are the maximum and minimum betweenness scores of all nodes in the network.

In this study, if TFs have a normalized betweenness centrality score > 0.1, then they are identified as potential TFs. Similarly, a set of light-responsive genes associated with potential TFs was prioritized in relation to growth and development as well as cordycepin and carotenoid biosynthetic pathways. Through this analysis, cytoHubba as a plugin in Cytoscape [44] was used.

### 3.4. Molecular Docking Simulation of the CmWC-1 with Its Potential Protein Targets

The 3D-structure homology model of CmWC-1 (CCM_01180) and all target candidates were predicted using the SWISS-MODEL webserver (https://swissmodel.expasy.org/interactive) (accessed on 7 August 2022), and the suggested templates with the highest similarity scores were selected for structural prediction. The resulting PDB files from SWISS-MODEL prediction were used for the template-based docking between CmWC-1 and all candidate targets using a state-of-the-art package: HDOCK [34] (http://hdock.phys.hust.edu.cn/) (accessed on 10 August 2022). The top-ranked model of each simulation was considered for the comparative analysis of binding affinity, stability and confidence scores among all targets.

## 4. Conclusions

This study combined an eGRN and transcriptome data analyses of *C. militaris* under light and dark conditions to identify light-responsive genes and potential transcription factors (TFs) involved in the growth, development, and biosynthesis of cordycepin and carotenoids. We identified five potential TFs—homeobox TF (CCM_07504), FlbC (CCM_04849), FlbB (CCM_01128), C6 zinc finger TF (CCM_05172), and mcrA (CCM_06477)—and ten genes regulated within a light-responsive subnetwork that are crucial for growth, development, and secondary metabolite production in *C. militaris*. Additionally, molecular docking analysis suggested that two novel TFs, CCM_05727 and CCM_06992, are promising candidates for interacting with the primary light-responsive protein CmWC-1. This information could lead to more effective strategies for cultivating and optimizing high-value metabolites in *C. militaris* and related fungi in industrial biotechnology.

## Figures and Tables

**Table 1:** Growth characteristics and production of cordycepin and carotenoids.

Conditions	Biomass (g kg^−1^)	Cordycepin Content (mg g^−1^ Cell Mass)	Carotenoid Content (mg g^−1^ Cell Mass)
Dark	57.857 ± 4.67	13.014 ± 0.04	0.092 ± 0.05
Light	74.376 ± 3.35 *	23.294 ± 0.80 *	1.916 ± 0.09 *

Values are presented as mean ± standard deviation. * Indicates statistically significant difference (*p* ≤ 0.05) from dark condition.

**Table 2:** Summary of RNA-seq data mapping to *C. militaris* CM01 genome.

Sample	Total Clean Reads (M)	GC (%)	Uniquely Mapped Reads (M)	Percentage of Uniquely Mapped Reads
D-1	24.88	56.00	23.81(95.71%)	95.71
D-2	19.96	56.00	19.05 (95.48%)	95.46
L-1	22.25	56.00	21.32 (95.84%)	95.84
L-2	24.48	57.00	23.50 (95.99%)	95.99
Average	22.89 ± 1.76	56.25 ± 0.39	21.92 ± 1.71	95.76 ± 0.19

**Table 3:** The potential light-responsive TFs of *C. militaris*.

Gene ID	TFs	DEGs	Number of Regulated Genes by TFs	Normalized Betweenness Score
CCM_07504	Homeobox transcription factor	Up	186	1.00
CCM_04849	C2H2 ZF transcription factor (FlbC)	Down	21	0.11
CCM_01128	bZIP-type transcription factor (FlbB)	Down	20	0.12
CCM_05172	C6 zinc finger domain containing protein	Up	16	0.10
CCM_06477	C6 transcription factor (mcrA)	Up	14	0.13

Note: DEGs—up means upregulation and down means downregulation.

**Table 4:** The docking score, confidence score, and conformational stability (RMSD) of potential cmWC-1 targets of *C. militaris*.

Ligands (Target Proteins)	CCM_ 00151	CCM_ 07874	CCM_ 04852	CCM_ 07378	CCM_ 05727	CCM_ 04347	CCM_ 06992	CCM_ 00072	CCM_ 01336
**Docking Score**	−273.95	−222.17	−242.87	−239.55	−330.6	−254.37	−289.5	−301.36	−262.15
**Confidence Score**	0.923	0.809	0.865	0.857	0.974	0.890	0.942	0.954	0.904
**Ligand RMSD (Å)**	65.63	56.07	45.19	44.50	59.91	59.36	38.27	79.80	79.58
**Template receptor (PDB ID)**	4F3L	4F3L	4F3L	4F3L	4F3L	4F3L	4F3L	4F3L	4F3L
**Template ligand (PDB ID)**	1DNP	2L2F	5A8I	3P8A	3GIA	5D3Q	-	4M4X	2LDU

## Data Availability

The original contributions presented in the study are included in the article/Supplementary Material, further inquiries can be directed to the corresponding authors.

## Supplementary Materials

The following supporting information can be downloaded at: https://www.mdpi.com/article/10.3390/ijms251910516/s1.
