# Supplementary material for: Expanded Gene Regulatory Network Reveals Potential Light-Responsive Transcription Factors and Target Genes in Cordyceps militaris
Source: Int J Mol Sci. 2024 Sep 29;25(19):10516. doi: 10.3390/ijms251910516 (PMC11476991; doi:10.3390/ijms251910516)
Supplement: Supplementary file 1 [file ijms-25-10516-s001.zip › ijms-3189819-supplementary.pdf]

Supplementary Table S1: List of 256 target proteins/genes from the light responsive TFs-genes interactive network between the 5 key transcription factors and their target genes (nodes)

Key light- responsive TFs	Target proteins/genes (nodes)
CCM_07504	CCM_08684
CCM_07504	CCM_01530
CCM_07504	CCM_03388
CCM_07504	CCM_01149
CCM_07504	CCM_03216
CCM_07504	CCM_02795
CCM_07504	CCM_05682
CCM_07504	CCM_05419
CCM_07504	CCM_02283
CCM_07504	CCM_04971
CCM_07504	CCM_09329
CCM_07504	CCM_02030
CCM_07504	CCM_04659
CCM_07504	CCM_04757
CCM_07504	CCM_04388
CCM_07504	CCM_01688
CCM_07504	CCM_03140
CCM_07504	CCM_01239
CCM_07504	CCM_08282
CCM_07504	CCM_01240
CCM_07504	CCM_02131
CCM_07504	CCM_00597
CCM_07504	CCM_09537
CCM_07504	CCM_09576
CCM_07504	CCM_09297
CCM_07504	CCM_07508
CCM_07504	CCM_09286
CCM_07504	CCM_08155
CCM_07504	CCM_06332
CCM_07504	CCM_06012
CCM_07504	CCM_06510
CCM_07504	CCM_06069
CCM_07504	CCM_05547
CCM_07504	CCM_05016
CCM_07504	CCM_08115
CCM_07504	CCM_05265
CCM_07504	CCM_04367
CCM_07504	CCM_04234
CCM_07504	CCM_03831

CCM_07504	CCM_02395
CCM_07504	CCM_07013
CCM_07504	CCM_06990
CCM_07504	CCM_01743
CCM_07504	CCM_01405
CCM_07504	CCM_02608
CCM_07504	CCM_02560
CCM_07504	CCM_02444
CCM_07504	CCM_02445
CCM_07504	CCM_02405
CCM_07504	CCM_05923
CCM_07504	CCM_02258
CCM_07504	CCM_08011
CCM_07504	CCM_09527
CCM_07504	CCM_01647
CCM_07504	CCM_09526
CCM_07504	CCM_09351
CCM_07504	CCM_01312
CCM_07504	CCM_09043
CCM_07504	CCM_07056
CCM_07504	CCM_05253
CCM_07504	CCM_06555
CCM_07504	CCM_08117
CCM_07504	CCM_05153
CCM_07504	CCM_07933
CCM_07504	CCM_00622
CCM_07504	CCM_05953
CCM_07504	CCM_04977
CCM_07504	CCM_06820
CCM_07504	CCM_05676
CCM_07504	CCM_06924
CCM_07504	CCM_05388
CCM_07504	CCM_05035
CCM_07504	CCM_04059
CCM_07504	CCM_03378
CCM_07504	CCM_03848
CCM_07504	CCM_03995
CCM_07504	CCM_03952
CCM_07504	CCM_03482
CCM_07504	CCM_00770
CCM_07504	CCM_03175
CCM_07504	CCM_03112
CCM_07504	CCM_07655

CCM_07504	CCM_08705
CCM_07504	CCM_08592
CCM_07504	CCM_01643
CCM_07504	CCM_08329
CCM_07504	CCM_01150
CCM_07504	CCM_05721
CCM_07504	CCM_05560
CCM_07504	CCM_00792
CCM_07504	CCM_05702
CCM_07504	CCM_00290
CCM_07504	CCM_05701
CCM_07504	CCM_05395
CCM_07504	CCM_09664
CCM_07504	CCM_09375
CCM_07504	CCM_02566
CCM_07504	CCM_04909
CCM_07504	CCM_02567
CCM_07504	CCM_04782
CCM_07504	CCM_08918
CCM_07504	CCM_04531
CCM_07504	CCM_08603
CCM_07504	CCM_08106
CCM_07504	CCM_08440
CCM_07504	CCM_07471
CCM_07504	CCM_06844
CCM_07504	CCM_00908
CCM_07504	CCM_00997
CCM_07504	CCM_00708
CCM_07504	CCM_02565
CCM_07504	CCM_03444
CCM_07504	CCM_01639
CCM_07504	CCM_02626
CCM_07504	CCM_00991
CCM_07504	CCM_00343
CCM_07504	CCM_04876
CCM_07504	CCM_06458
CCM_07504	CCM_03730
CCM_07504	CCM_04871
CCM_07504	CCM_08556
CCM_07504	CCM_03168
CCM_07504	CCM_08002
CCM_07504	CCM_09207
CCM_07504	CCM_00729

CCM_07504	CCM_04890
CCM_07504	CCM_09350
CCM_07504	CCM_04399
CCM_07504	CCM_04527
CCM_07504	CCM_08802
CCM_07504	CCM_08832
CCM_07504	CCM_02558
CCM_07504	CCM_07917
CCM_07504	CCM_06584
CCM_07504	CCM_06306
CCM_07504	CCM_06451
CCM_07504	CCM_00196
CCM_07504	CCM_05570
CCM_07504	CCM_09534
CCM_07504	CCM_09393
CCM_07504	CCM_05136
CCM_07504	CCM_04892
CCM_07504	CCM_02392
CCM_07504	CCM_09016
CCM_07504	CCM_04589
CCM_07504	CCM_04516
CCM_07504	CCM_04434
CCM_07504	CCM_08189
CCM_07504	CCM_08213
CCM_07504	CCM_08083
CCM_07504	CCM_09483
CCM_07504	CCM_08039
CCM_07504	CCM_09211
CCM_07504	CCM_07839
CCM_07504	CCM_01147
CCM_07504	CCM_01706
CCM_07504	CCM_00827
CCM_07504	CCM_07920
CCM_07504	CCM_06614
CCM_07504	CCM_09684
CCM_07504	CCM_09099
CCM_07504	CCM_06572
CCM_07504	CCM_06613
CCM_07504	CCM_07138
CCM_07504	CCM_06001
CCM_07504	CCM_06603
CCM_07504	CCM_04678
CCM_07504	CCM_07260

CCM_07504	CCM_06500
CCM_07504	CCM_06438
CCM_07504	CCM_09504
CCM_07504	CCM_06365
CCM_07504	CCM_08946
CCM_07504	CCM_07595
CCM_07504	CCM_05677
CCM_07504	CCM_03644
CCM_07504	CCM_04867
CCM_07504	CCM_05211
CCM_07504	CCM_05172
CCM_07504	CCM_03438
CCM_07504	CCM_01740
CCM_07504	CCM_00306
CCM_07504	CCM_04286
CCM_07504	CCM_09071
CCM_07504	CCM_03994
CCM_07504	CCM_09072
CCM_04849	CCM_09583
CCM_04849	CCM_09534
CCM_04849	CCM_09244
CCM_04849	CCM_08952
CCM_04849	CCM_09194
CCM_04849	CCM_08802
CCM_04849	CCM_08041
CCM_04849	CCM_07882
CCM_04849	CCM_07881
CCM_04849	CCM_07855
CCM_04849	CCM_05702
CCM_04849	CCM_05704
CCM_04849	CCM_05701
CCM_04849	CCM_05494
CCM_04849	CCM_05097
CCM_04849	CCM_04727
CCM_04849	CCM_02865
CCM_04849	CCM_01567
CCM_04849	CCM_01568
CCM_04849	CCM_00908
CCM_04849	CCM_00002
CCM_01128	CCM_07106
CCM_01128	CCM_06957
CCM_01128	CCM_05771
CCM_01128	CCM_05701

CCM_01128
CCM_01128
CCM_01128
CCM_01128
CCM_01128
CCM_01128
CCM_01128
CCM_01128
CCM_01128
CCM_01128
CCM_01128
CCM_01128
CCM_01128
CCM_01128
CCM_01128
CCM_01128
CCM_05172
CCM_05172
CCM_05172
CCM_05172
CCM_05172
CCM_05172
CCM_05172
CCM_05172
CCM_05172
CCM_05172
CCM_05172
CCM_05172
CCM_05172
CCM_05172
CCM_05172
CCM_06477
CCM_06477
CCM_06477
CCM_06477
CCM_06477
CCM_06477
CCM_06477
CCM_06477
CCM_06477
CCM_06477
CCM_06477
CCM_06477

CCM_05702
CCM_05136
CCM_04876
CCM_04420
CCM_04072
CCM_04071
CCM_03466
CCM_03737
CCM_03182
CCM_02974
CCM_02148
CCM_01969
CCM_01997
CCM_01740
CCM_00331
CCM_08804
CCM_02764
CCM_03587
CCM_03878
CCM_03900
CCM_03901
CCM_05061
CCM_05547
CCM_06214
CCM_06332
CCM_06441
CCM_06511
CCM_07881
CCM_07882
CCM_08092
CCM_09464
CCM_09534
CCM_09037
CCM_09485
CCM_08879
CCM_06603
CCM_06063
CCM_05372
CCM_04907
CCM_04892
CCM_04264
CCM_03324
CCM_02038

CCM_06477	CCM_01064
CCM_06477	CCM_00173
